# Building Accurate Intracellular Polarity Maps through Multiparametric Microscopy

**DOI:** 10.3390/mps3040078

**Published:** 2020-11-11

**Authors:** M. Carmen Gonzalez-Garcia, Pilar Herrero-Foncubierta, Emilio Garcia-Fernandez, Angel Orte

**Affiliations:** 1 Departamento de Fisicoquimica, Facultad de Farmacia, University of Granada, 18071 Granada, Spain; mcarmeng@ugr.es (M.C.G.-G.); pilarhf@ugr.es (P.H.-F.); emiliogf@ugr.es (E.G.-F.); 2 Departamento de Quimica Organica, Facultad de Ciencias, University of Granada, 18071 Granada, Spain

**Keywords:** biosensing, cellular microenvironment, fluorescence imaging, fluorescence lifetime imaging microscopy (FLIM), lifetime, acridones, solvatochromism

## Abstract

The precise knowledge of intracellular polarity, a physiological parameter that involves complex and intertwined intracellular mechanisms, may be relevant in the study of important diseases like cancer or Alzheimer’s. In this technical note, we illustrate our recently developed, accurate method for obtaining intracellular polarity maps employing potent fluorescence microscopy techniques. Our method is based on the selection of appropriate luminescent probes, in which several emission properties vary with microenvironment polarity, specifically spectral shifts and luminescence lifetime. A multilinear calibration is performed, correlating polarity vs. spectral shift vs. luminescence lifetime, to generate a powerful and error-free 3D space for reliable interpolation of microscopy data. Multidimensional luminescence microscopy is then used to obtain simultaneously spectral shift and luminescence lifetime images, which are then interpolated in the 3D calibration space, resulting in accurate, quantitative polarity maps.

## 1. Introduction

In this technical note, we describe a recently developed, accurate method for sensing intracellular polarity (dipolarity), using multiparametric fluorescence microscopy. Sensing microenvironment polarity is of especial interest for intracellular studies, since many different cellular processes, such as recognition interactions and biochemical reaction kinetics that include metabolism, membrane transport, immune response, degradation of cellular components or cellular aging, among others, are controlled by the general (such as hydrophobic interactions) and specific features (such as hydrogen bonding or sulfide bridges) of the environment [1]. Likewise, disease-related intracellular alterations may cause changes in microenvironment polarity—for instance, due to anomalous metabolism in some diseases such as cancer [2,3] or Alzheimer’s disease [4,5,6].

To date, several methods to study intracellular polarity, based on luminescent probes, have been reported. Most of these methods are based on ratiometric sensors, using dual-channel fluorescence microscopy. However, the ubiquitous presence of cellular autofluorescence and other sources of interference prevents these methods from quantitatively and accurately determining intracellular polarity, and they are merely capable of reporting on relative polarity changes [3,4,7,8,9,10,11].

Herein, we present a new methodology aimed at exploring the local polarity of heterogeneous media, and particularly, we applied it for cell imaging in order to obtain an intracellular polarity map [12]. For this purpose, we employ the photophysical information provided by a fluorophore that is extremely sensitive to the local polarity environment. Our method overcomes the limitations of conventional ratiometric approaches by taking advantage of multidimensional fluorescence microscopy to perform a reliable and robust multilinear calibration that allows quantitative determination of intracellular polarity.

## 2. Experimental Design

### 2.1. Requirements of Luminescence Probes

The design of suitable/tailored solvatochromic luminophores is the crucial starting point and, perhaps, the most important step for this method. The method requires a luminescent probe that is extremely sensitive to microenvironment polarity, with an impact on several photophysical parameters, such as the emission maxima, intensity and luminescence lifetime, while being robust and insensitive enough to other chemical parameters commonly found in the cell interior, such as the pH or the presence of specific cations, anions and other potential quenchers.

Crucially, one of the most important factors that prevents quantitative sensing inside cells is the contribution of cellular autofluorescence emission, which may cause drastic systematic errors in quantitative determinations [13]. A very sought-after feature for luminescent probes to avoid cellular autofluorescence is the emission in the far-red and near-infrared (NIR) regions of the spectra [14]. However, far-red or NIR dyes with suitable sensing capabilities are not always available. Another strategy for the effective filtering of cellular autofluorescence as a source of systematic interference is the use of luminophores that exhibit long values of the luminescence lifetime, τ. Specifically, the emitted signal from fluorophores with τ values higher than 6 ns can be effectively discriminated from autofluorescence, using a time-gated analysis [15]. Alternatively, phosphorescent luminophores provide a long-lived signal that allows for filtering out all sources of fluorescent interferences [15,16,17].

**Figure 1:**
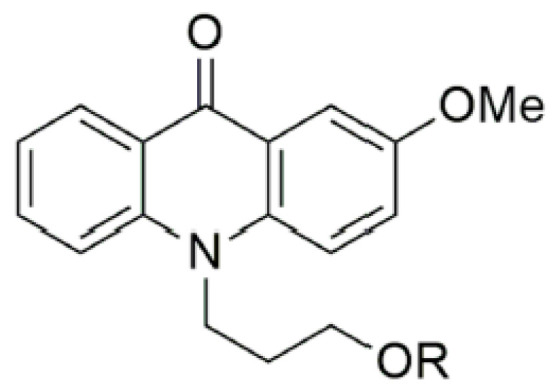
Structure of N-substituted acridones as suitable probes for intracellular polarity maps.

In the development of this method, we explored the use of N-substituted-2-methoxyacridones (Figure 1). This family of fluorophores exhibits desirable features such as long fluorescence lifetime (>10 ns), marked solvatochromism [12] and pH insensitivity in the near-neutral range [18]. In particular, depending on substituent R-, we employed dyes **1** (R = H), **2** (R = Me) and **3** (R = Ph). The family of N-substituted-2-methoxyacridone derivatives has the advantage that the R position does not affect the main photophysical properties of the fluorophore, so that tailored modifications can be included—for instance, for specific targeting of cellular organelles.

Importantly, the use of N-substituted-2-methoxyacridone acridones is not a compulsory demand. Other solvatochromic dyes, with long luminescence lifetimes, such as naphthamilide or quinolimide dyes [19,20], may be valid candidates for implementing the method.

### 2.2. Requirements of Equipment

Regarding the necessary equipment to carry out this method, a dual-channel fluorescence lifetime imaging microscope (FLIM) is the main instrument to be employed. The method to build up accurate intracellular polarity maps is based on a multilinear calibration of, at least, two independent parameters characterizing the probe emission. Hence, the confocal fluorescence microscope must be equipped with time-resolved, photon counting electronics for FLIM imaging and, at least, two different detection channels with different filters to collect two independent color images, aiming to reconstruct ratiometric images.

In this development, we employed a dual-channel FLIM microscope (MicroTime 200, PicoQuant GmbH, Berlin, Germany), equipped with a 375 nm pulsed excitation laser (LDH-375, PicoQuant GmBH, Berlin, Germany). The collected emission fluorescence was separated into two detection channels, after passing through a 450/40 or 500/40 bandpass filter, and detected in two single-photon avalanche diodes (SPCM-AQR-14, Perkin Elmer, Waltham, MA, USA).

Nevertheless, other configurations may be possible to implement the method. For instance, instead of dual-channel detection, spectral imaging or hyperdimensional imaging can be employed [21,22].

## 3. Procedure

### 3.1. Step 1: Calibration with Solvent Polarity

Fluorescent properties of the probe need to be tested in selected solvents that mimic the heterogeneity of the media we are interested in exploring. Therefore, solvents with relevant and different polarity parameters according to, for example, Marcus or Catalan [23,24] polarity scales must be chosen: high and low polar solvents, hydrogen donor and/or acceptor solvents, etc. In particular, we suggest the use of the E_T_30 solvent polarity scale, defined as the difference in energy of the excited- and ground-state levels of the dye betaine 30 [25], because this parameter involves both general (polarity) and specific (hydrogen bonding) solvent effects.

**Figure 2:**
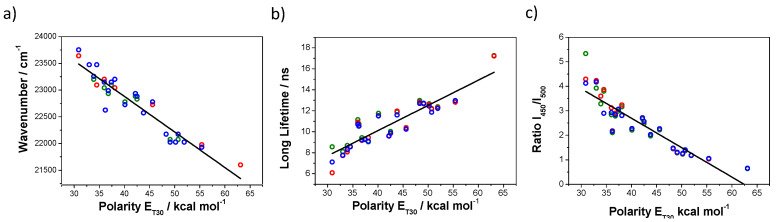
Dependence with the E_T_30 (kcal mol^–1^) value of (**a**) wavenumber of emission maximum peak, (**b**) long lifetime and (**c**) intensity ratio between emission at 450 nm and emission at 500 nm (*I*_450_/*I*_500_) of acridones **1** (green), **2** (red) and **3** (blue).

Firstly, the fluorophore’s emission features must be investigated using high-quality steady-state and time-resolved spectrofluorimeters. In our case, we employed a Jasco FP8300 steady-state spectrofluorimeter (Jasco, Tokyo, Japan) and a PicoQuant FluoTime 200 time-resolved fluorimeter. Then, the most relevant photophysical parameters, especially sensitive to solvent polarity in terms of E_T_30, are selected. In the case of acridones **1**–**3**, the fluorescence emission maxima, intensity ratios and lifetimes showed marked dependency with the E_T_30 values (Figure 2).

If different luminophore probes are employed in the method, other potential photophysical features that may be sensitive to the microenvironment are fluorescence anisotropy or excited-state kinetics, to mention a few, and they can be also employed to construct the calibration curve.

### 3.2. Step 2: Multiparametric Calibration Curve Fitting

Once the most relevant photophysical parameters are selected and their linear correlation with solvent polarity is confirmed, a multi-linear calibration can be performed, according to the following equation:

(1) $$E_{T}30\left(/\mathrm{kcal}\cdot {\mathrm{mol}}^{-1}\right)=a+b\cdot x+c\cdot y+d\cdot z+\ldots$$

where *a*, *b*, *c*, *d*… are the fitting parameters and *x*, *y*, *z*… are the chosen photophysical properties of the dye.

**Figure 3:**
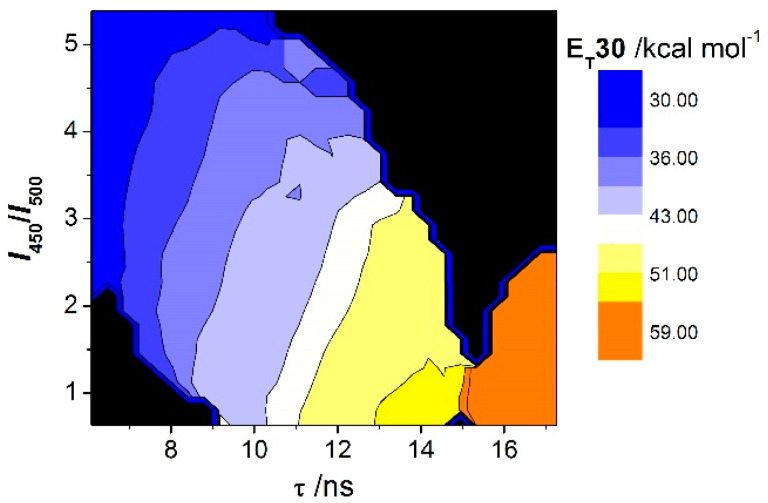
Multiparametric calibration of E_T_30 values in a 3D space employing lifetime and time-gated intensity ratio *I*_450_/*I*_500_ from dyes **1**–**3**.

In the development of this method, we focused on the dependence of E_T_30 on two parameters of the N-substitued-2-methoxyacridones: the intensity ratio (*I*_450_/*I*_500_) and fluorescence lifetime (τ); see Equation (1). A 3D correlation plot can be constructed (Figure 3) showing the multiparametric dependence of the fluorophore properties on the polarity E_T_30 scale.

(2) $$E_{T}30\left(/\mathrm{kcal}\cdot {\mathrm{mol}}^{-1}\right)=a+b\cdot \tau \left(/ns\right)+c\cdot \left[I_{450}/I_{500}\right]$$

In the case of acridones **1**–**3**, the photophysical properties were comparable, since the different radical R- does not affect the fluorophore moiety. Hence, a global calibration for the three dyes was performed, and we obtained the multi-linear Equation (3):

(3) $$E_{T}30\left(/\mathrm{kcal}\cdot {\mathrm{mol}}^{-1}\right)=36.24+1.52\cdot \tau \left(/ns\right)-4.18\cdot \left[I_{450}/I_{500}\right]$$

We performed this calibration using Microsoft Excel’s (Microsoft, Redmond, WA, USA) LINEST function, which allows powerful multivariate analysis. An important step is subsequently judging the goodness of fitting. This can be achieved by regression coefficient analysis (*r*^2^ value was 0.87 for Equation (3)) and by the *F*-test. The LINEST function provides the *F*-statistic value of the analysis. This value can be compared to the critical *F* value from the *F*(*v*_1_, *v*_2_) distribution to assess the likelihood of a larger *F* value occurring by chance, at a certain probabilistic confidence level. The critical *F*(*v*_1_, *v*_2_) value, at a certain probability level α, can be obtained either from published tables [26] or by using the F.INV.RT function in Excel, where *v*_1_ = *N* − *df* − 1 and *v*_2_ = *df*, *N* is the number of datapoints, and *df* represents the degrees of freedom. For the fit in Equation (3), the obtained *F* value was 399. The critical *F*(2,121) at a confidence level of 0.1% (α = 0.001, *N* = 124 points, *df* = 121) is 7.3. Obviously, the substantial difference between the *F* value from the fit and the critical value ensures that it is extremely unlikely that such a large *F* value occurred by chance.

### 3.3. Step 3: Multiparametric Fluorescence Imaging

Once we have calibrated our probe, we can introduce it in the media we are interested in interrogating—for example, to explore intracellular polarity. To apply the method described herein, it is necessary to use a microscopy technique able to acquire simultaneously all the sensitive photophysical parameters chosen for the calibration. In this particular case, a multiparametric fluorescence imaging technique is of special relevance, such FLIM, able to obtain a multilayer image containing intensity and lifetime information with spectral and spatial resolution.

**Figure 4:**
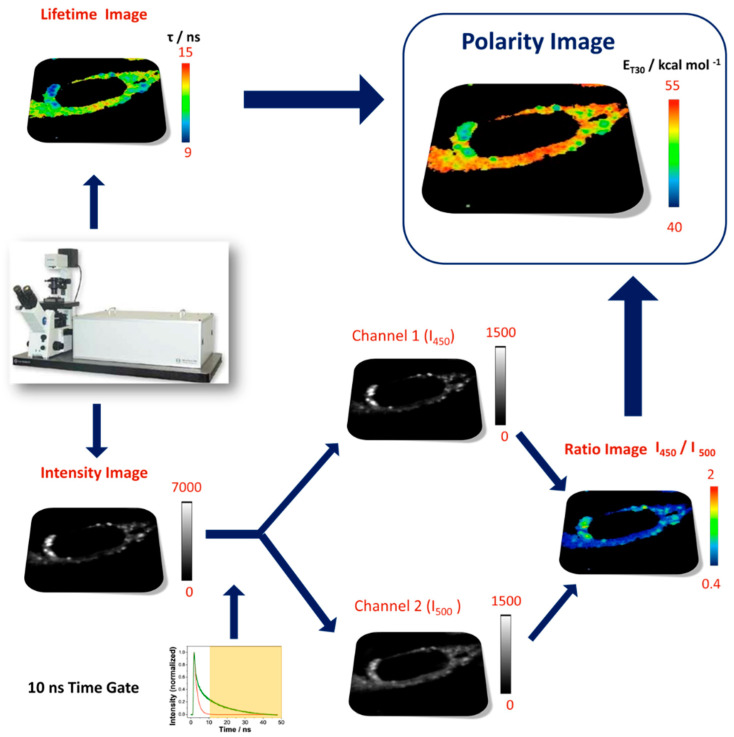
Scheme of the method to obtain accurate intracellular dipolarity images, in terms of the E_T_30 parameter, through multiparametric fluorescence microscopy.

In this method, multichannel FLIM acquisition provides intensity and lifetime information for two different spectral bandwidths in a 2D image composed of thousands of pixels; see Figure 4. We employed three layers of information from each FLIM image: intensity in the 450/40 nm band (layer 1, *I*_450_), intensity in the 500/40 nm band (layer 2, *I*_500_) and the average lifetime in both channels (layer 3, τ). Intensities at both detection channels can be used to obtain the ratiometric parameter (*I*_450_/*I*_500_), independent of probe concentration and laser power.

### 3.4. Step 4: Building Polarity Maps

To reconstruct accurate and quantitative estimations of microenvironment polarity from the multiparametric FLIM microscopy data described above, an important previous step lies in the removal of all potential sources of undesired fluorescent interferences, such as cellular autofluorescence, by using time-gated filtered intensity images. We perform a previous time-gate filtering of layers 1 and 2 to remove autofluorescence from cells and, subsequently, calculate an interference-free ratiometric image. The contribution of cellular autofluorescence would cause systematic errors in the intensity ratio; hence, this point is crucial for reliable polarity quantification and justifies the requirement of fluorescent probes with long fluorescence lifetimes as well as a time-resolved microscopy technique, such as FLIM. The time-gated filtering removes all the short-lived emissive components [15], accounting only for long-lived photons from the probe. Likewise, application of the time-gating approach makes it unnecessary to perform any background correction to the images, since all the photons collected in that specific time window are emitted from the sensing fluorophore. It is important to note that if a common fluorophore were employed, with a lifetime in the order of cellular autofluorescence (2–3 ns), effective time-gated filtering would not be possible.

In addition, to appropriately use the calibration in Equation (2) in microscopy experiments—in particular, using an intensity-based parameter—it is important to consider the sensitivity dependence to radiation of different wavelengths of the fluorimeter and microscope detectors. Steady-state fluorimeters are usually equipped with photomultiplier tubes as detectors, whereas multidimensional and FLIM microscopes may be furnished with EMCCD (Electron-multipliying charge-coupled device) cameras, photomultiplier tubes, single-photon avalanche photodiodes (SPADs) and/or hybrid detectors. Each type of detector has a different spectral response, which is important to consider for the appropriate interpolation of experimental data in the multi-linear calibration in Equation (2). This is especially important in the near-UV and the red region of the spectrum, to which many of the listed detectors show reduced sensitivity. As an example, the SPCM-AQR-14 SPAD in our microscope exhibits a sensitivity maximum at 700 nm, whereas the detection efficiency decreases by half at 450 nm. To perform such correction, the recorded intensity on a detection channel of the microscope must be multiplied by a factor accounting for the differences in the detectors’ spectral sensitivity. The spectral response curves of the detectors are normally supplied by the detector manufacturer. To obtain the correction factor, the detector’s response curve is integrated across the detection band for each channel (depending on the bandpass filter used), resulting in a specific area value. The correction factor is then obtained by dividing the area value of the calibration detector by the area value of the microscope detector. It is worth mentioning that this correction is only required for intensity measurements, whereas the fluorescence lifetimes are intensity-independent and, hence, no further corrections are required for this parameter.

In our particular case, the *I*_450_/*I*_500_ ratio was corrected to account for the different spectral sensitivity of the microscope SPAD detectors (acquisition detector) compared to the fluorimeter photomultiplier tube (calibration detector) for the two emission bands, *I*_450_ (450 ± 20 nm) and *I*_500_ (500 ± 20 nm).

Once these corrections are implemented, we have built the lifetime τ image as well as the time-gated *I*_450_/*I*_500_ ratio image (Figure 4). These images, in matrix form, are then brought into Equation (3) to obtain the corresponding E_T_30 polarity image (Figure 4).

## 4. Method Validation—Intracellular Polarity Maps in Different Cellular Microenvironments

**Figure 5:**
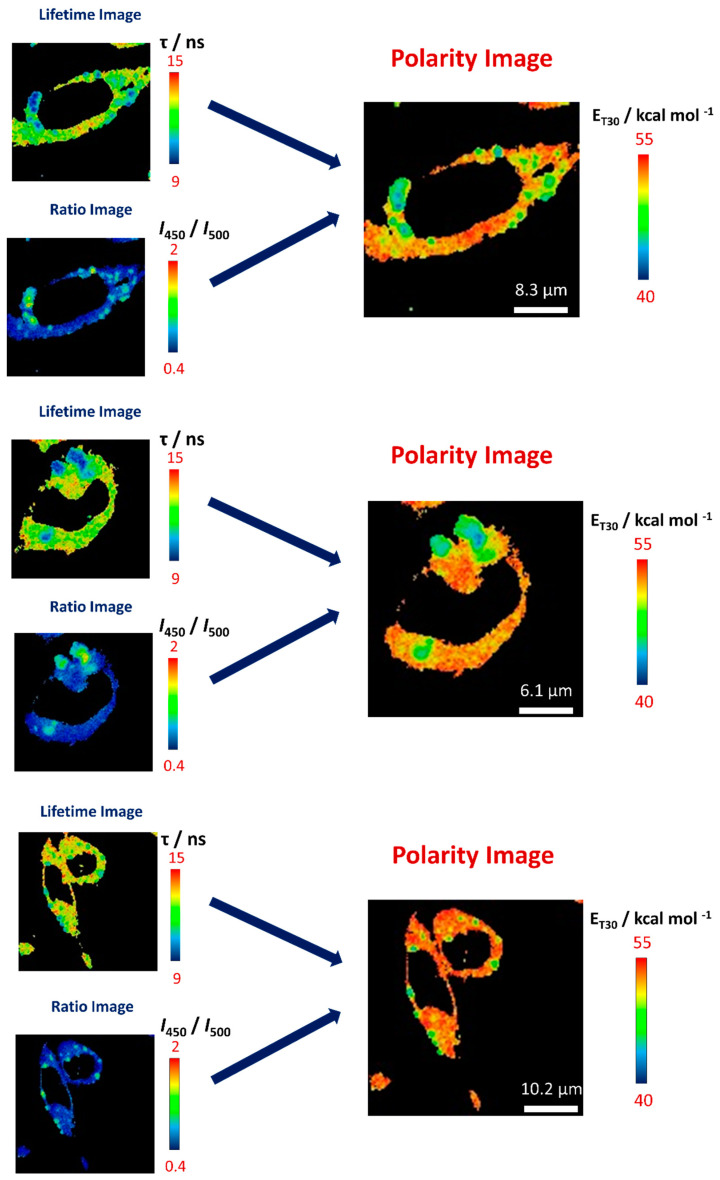
Examples of lifetime, time-gated *I*_450_/*I*_500_ ratio and reconstructed E_T_30 polarity images of dye **3** in CCD-18Co cells, using the method herein described.

We employed this methodology to obtain intracellular polarity maps of different types of cells, clearly pointing out the heterogeneous polarity inside cells (Figure 5). CCD-18Co cells provided by Cell Culture Facility of the University of Granada were incubated with dye **3** for a final concentration of 0.3 µM following the procedure explained elsewhere [1]. As described in our previous work, this methodology in conjunction with acridone derivative probes allows for distinguishing different parts of cells, such as nucleus, cytosol, lysosomes, according to their polarity.

The versatility and general applicability of this method is an important feature to emphasize. The method can be easily adapted to use other type of luminophores as probes, as long as they fulfill the general feature of two orthogonal photophysical properties responding to microenvironment polarity. Likewise, even though we applied it to explore the intracellular environment, this method can be used in multipurpose approaches, such as the polarity of hydrogels, vesicles or heterogeneous matrixes, making it a promising and powerful tool to characterize the microenvironment of complex systems and to track potential changes in these systems with great accuracy.
